# Mobility overestimation due to gated contacts in organic field-effect transistors

**DOI:** 10.1038/ncomms10908

**Published:** 2016-03-10

**Authors:** Emily G. Bittle, James I. Basham, Thomas N. Jackson, Oana D. Jurchescu, David J. Gundlach

**Affiliations:** 1 National Institute of Standards and Technology, Engineering Physics Division, 100 Bureau Drive, MS 8120, Gaithersburg, Maryland 20899, USA; 2 Department of Physics, Wake Forest University, 1834 Wake Forest Road, Winston-Salem, North Carolina 27109, USA; 3 Department of Electrical Engineering, The Pennsylvania State University, 121 Electrical Engineering East, University Park, State College, Pennsylvania 16802, USA

## Abstract

Parameters used to describe the electrical properties of organic field-effect transistors, such as mobility and threshold voltage, are commonly extracted from measured current–voltage characteristics and interpreted by using the classical metal oxide–semiconductor field-effect transistor model. However, in recent reports of devices with ultra-high mobility (>40 cm^2^ V^−1^ s^−1^), the device characteristics deviate from this idealized model and show an abrupt turn-on in the drain current when measured as a function of gate voltage. In order to investigate this phenomenon, here we report on single crystal rubrene transistors intentionally fabricated to exhibit an abrupt turn-on. We disentangle the channel properties from the contact resistance by using impedance spectroscopy and show that the current in such devices is governed by a gate bias dependence of the contact resistance. As a result, extracted mobility values from d.c. current–voltage characterization are overestimated by one order of magnitude or more.

## Abstract (web summary)

Charge mobility, extracted from current–voltage curves, is an important parameter for evaluating the performance of organic field-effect transistors. Bittle *et al*. show that charge mobility can be overestimated by one order of magnitude due to the gate bias dependence of the charge injection process.

## Graphical abstract

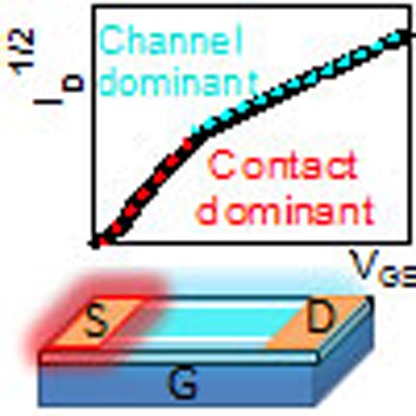

Organic semiconductors (OSCs) remain a topic of considerable interest for basic and applied research. As such, accurate electrical characterization and parameterization of physical properties which govern the device operation of light emitting diodes, field-effect transistors and photovoltaic cells is essential for continued device performance improvement and possible commercialization of organic semiconductor-based devices. Charge carrier mobility is one of several commonly cited physical properties, and describes charge motion under applied electric field. The organic field-effect transistor (OFET) is routinely used as a test structure for extracting mobility in addition to being a key element in circuits. The most commonly used method to evaluate OFET parameters such as field-effect mobility, *μ*, and threshold voltage, *V*_th_, is the classical metal-oxide–semiconductor field-effect transistor (MOSFET) model. This model is described for the two extreme modes of operation above threshold, |*V*_GS_|>*V*_th_|, in equations (1) and (2). For linear mode, |*V*_DS_|<|*V*_GS_−*V*_th_|,


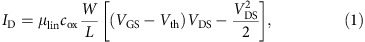


and for saturation mode |*V*_DS_|>|*V*_GS_−*V*_th_|,


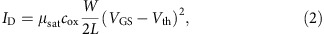


where *V*_GS_ is the gate voltage, *V*_th_ is the threshold voltage, *I*_D_ is the drain current, *V*_DS_ is the drain voltage, *μ*_lin_ and *μ*_sat_ are the linear and saturation mobility, respectively, *W* and *L* are the width and length of the transistor channel and *c*_ox_ is the oxide capacitance per unit area. In this paper, we illustrate how this model describing the device behaviour for idealized materials and interfaces that adhere to solid-state band theory can lead to severe inaccuracies in extracted parametric values when used to analyse non-ideal transistors.

We focus on a particular non-ideality in OFET transistor characteristics, shown in Fig. 1a, which appears in many high-mobility single-crystal FETs^1^,^2^,^3^, polymer^4^,^5^,^6^,^7^,^8^,^9^,^10^ and small molecule thin film transistors^11^,^12^. In this non-ideal case, *I*_D_ shows an abrupt change in slope as a function of *V*_GS_, whereas in the classical model *I*_D_ (*I*_D_^1/2^) is linear with *V*_GS_ in the linear (saturation) regime as defined in equations (1) and (2). This slope is regularly used to calculate mobility and extrapolate the threshold voltage. As reports of transistors with this non-ideal behaviour become more prevalent in the literature, yielding impressive mobility values, a detailed understanding of the source of the non-ideal behaviour and its impact on extracted figures of merit has become important. Here, we fabricate a transistor to exhibit non-ideal *I*_D_−*V*_GS_ characteristics and use impedance spectroscopy to disentangle the contact behaviour from the transistor channel behaviour to directly compare these measurements to d.c. I–V measurements made on the same OFET device. This comparison clarifies the impact that a non-ohmic contact can have on transistor behaviour, and by extension, on the mobility extracted from the I–V data. We show that the mobility can be overestimated by up to one order of magnitude in transistors with pronounced non-ideal current–voltage characteristics, an effect which arises from the gate-bias dependence of the contact resistance.

## Results

### Material and electrical considerations

**Figure 1:**
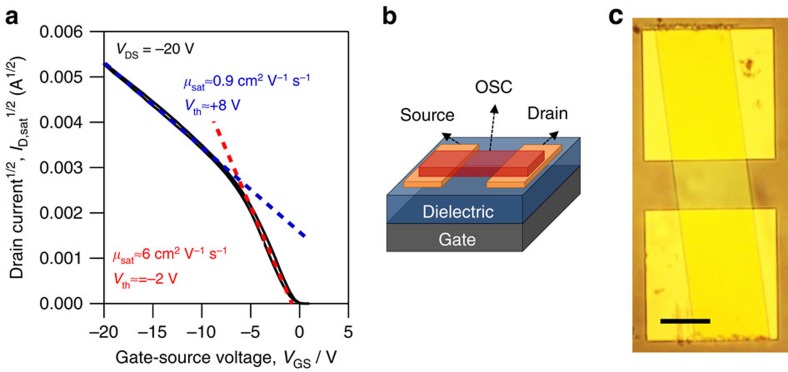
Current–voltage characteristics for a non-ideal transistor and transistor geometry. (**a**) Plot of the transfer characteristics in the saturation regime (*V*_DS_=−20 V) of a rubrene transistor exhibiting non-ideal characteristics. Fit lines in red and blue illustrate the ambiguity associated with characterizing OFETs. (**b**) Bottom gate/bottom contact OFET. (**c**) Image of transistor. Drain and source contact pairs are 250 × 250 μm squares and the rubrene active area is 140 μm wide × 100 μm long. Scale bar is 100 μm.

For this study, we characterized the electrical properties of single-crystal rubrene field-effect transistors fabricated in a bottom contact, bottom gate geometry (Fig. 1b). The bottom-contact geometry simplifies the electric field distribution and the parasitic gate to source/drain overlap capacitance, and eliminates charge transport in the out-of-plane direction in the OSC. Rubrene was chosen because its single crystals have been shown to exhibit nearly ideal transistor behaviour and are useful for fundamental studies^13^. Small molecule single crystals have lower molecular disorder and more straightforward transport pathways than polymer thin films, which involve a convolution of pi–pi and backbone transport. Furthermore, single crystals do not suffer from pronounced grain boundaries that can lead to large potential drops in the channel as in the case of polycrystalline thin films of small molecule semiconductors^14^,^15^. A micrograph of a completed field-effect transistor device is shown in Fig. 1c.

### Current–voltage characterization

The d.c. *I*_D_–*V*_GS_ characteristics for a device biased in the saturation regime with *V*_DS_=−20 V are plotted in Fig. 1a. For both saturation (|*V*_DS_|>|*V*_GS_−*V*_th_|) and linear (|*V*_DS_|<|*V*_GS_−*V*_th_|) regimes, the current–voltage characteristics change from high slope to low slope as the gate bias is increased from 0 V. The kink where the slope change happens, around −6 V in the saturation characteristics, allows us to define two regions: one at small gate bias (0>*V*_GS_>−6 V) with high slope and another at large gate bias (−20 V<*V*_GS_<−7 V) with low slope. This behaviour deviates from the ideal FET behaviour given by equations (1) and (2), where the slope is constant with *V*_GS_ (*I*_D_∝*V*_GS_ in the linear regime and *I*_D_^1/2^∝*V*_GS_ in the saturation regime.) The slope and intercept of linear fits to the *I*–*V* data provide an aggregate mobility and threshold voltage values when evaluated in the MOSFET model. In the saturation regime, these fits yield mobility of 6 cm^2^ V^−1^ s^−1^ and threshold voltage of −2 V at low gate bias, and 0.9 cm^2^ V^−1^ s^−1^ and 8 V at high gate bias. The extracted mobility differs by ∼6 × and *V*_th_ differs by 10 V.

**Figure 2:**
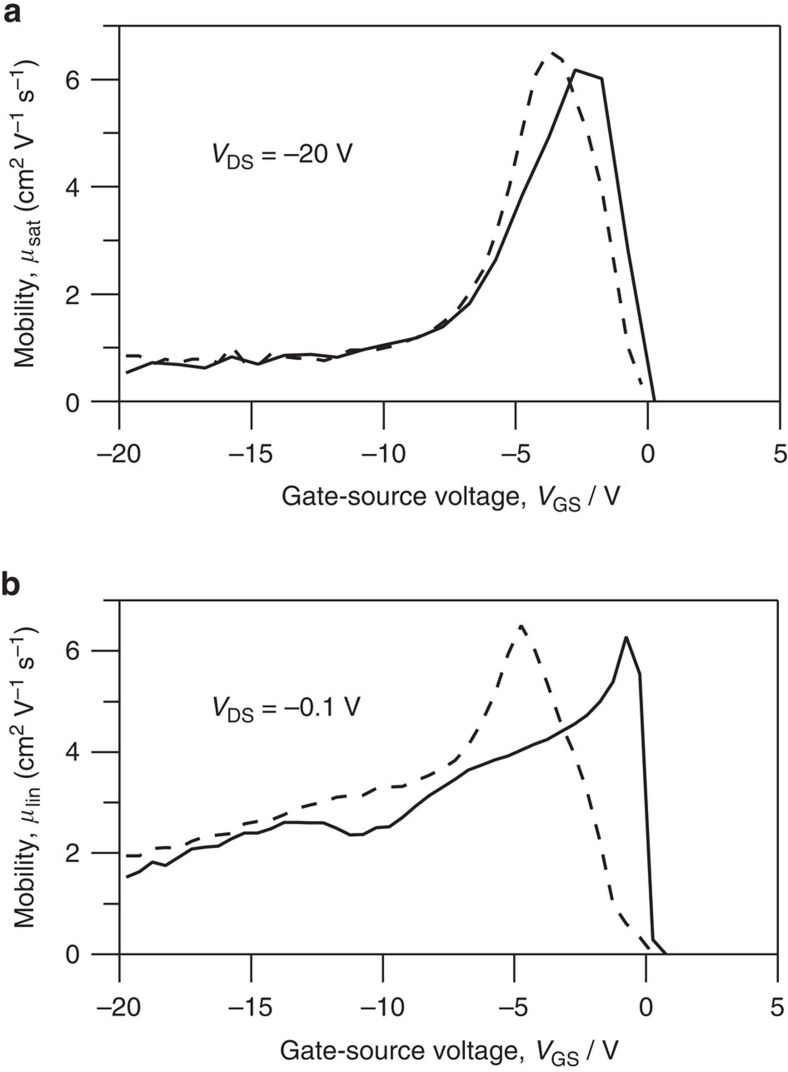
Overestimation of the mobility. ‘Mobility', as defined in equations (1) and (2), are plotted in the saturation (**a**) and linear (**b**) regimes as a function of gate bias, increasing bias direction in solid lines and reverse in dashed lines. Similar results were found for 30 transistors measured during the course of this study.

Mobility as a function of gate voltage was extracted using the MOSFET model for the saturation (*V*_DS_=−20 V) and linear (*V*_DS_=−0.1 V) regimes and is plotted in Fig. 2a,b. The differential mobility values extracted from the MOSFET model are nearly constant for −20 V<*V*_GS_<−10 V, but increase to a peak at *V*_GS_≈−5 V. Hysteresis, likely due to modest charge trapping in the transistor channel, is small. Forward and reverse sweeps show a comparable variation of the mobility with gate bias. The linear and saturation regime mobilities have similar magnitude and variation with gate bias, which creates ambiguity about the intrinsic transistor channel mobility.

**Figure 3:**
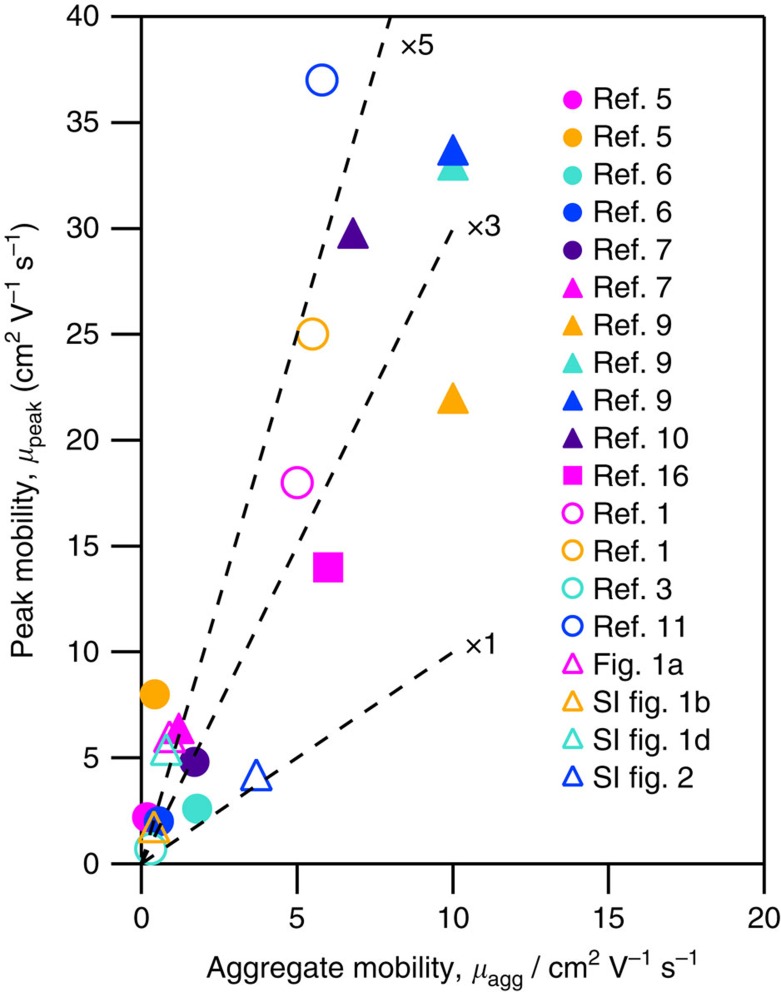
Prevalence of overestimation of the mobility. Our estimates of peak mobility, *μ*_peak_, at low gate bias and aggregate mobility, *μ*_agg_, calculated for higher gate bias using the MOSFET equations applied to hand fits of published data^1^,^3^,^5^,^6^,^7^,^9^,^10^,^11^,^16^. We also include our data from Fig. 1a and the Supplementary Figs (SI) 1b, 1d and 2. Polymers are given by filled symbols and small molecules are given by open symbols. Selected papers show a change from high to low slope in the transconductance data as gate bias is increased in either the saturation or linear regime for p-type conduction. Lines are guides to the eye, and show the ratio of peak to high gate bias values.

In several devices we fabricated using nominally the same processing method the discrepancy in the mobility was as large as 14 × . We have included two examples in Supplementary Fig. 1. For contrast, we have also included a sample prepared on platinum contacts, which shows nearly ideal behaviour in Supplementary Fig. 2. We have reexamined data from several references^1^,^3^,^5^,^6^,^7^,^9^,^10^,^11^,^16^ with a similar discrepancy between the high and low gate bias regions by applying equations (1) and (2) to our estimates from the slopes of published *I*–*V* characteristics. The comparison plotted in Fig. 3 show that for most of the data examined, the peak mobility is ∼3 × to 5 × of the aggregate mobility calculated at high gate bias; with largest disparity being 18 × . It is important to note that the mobility values calculated using the MOSFET model are scalar fit parameters and not intrinsic material parameters. We made an effort in our literature search to include data on polymers, small molecules, as well as semiconductors with different band gaps, but have found no correlation between this behaviour and material type.

Explanations for this non-ideal behaviour vary in the literature. Some have proposed that at low gate bias, the accumulation layer is not tightly confined to the interface and extends into the bulk where there is less effect of the disorder associated with the OSC/dielectric interface. At higher gate bias, the charges become confined to the interface at high gate fields and mobility decreases due to the increased disorder^1^, charges become trapped in the gate dielectric at high field^17^ or that the high carrier density causes Coulombic interactions between the charges^18^. Other reports have proposed that a high contact resistance restricts current at high gate bias when the channel and contact resistance become comparable^19^. However, this latter interpretation is not consistent with observations by several other groups who report a decrease in contact resistance with increasing gate bias^20^,^21^. Researchers studying ambipolar operation have speculated that this effect could be due to negative trap filling in low band gap materials^16^, which we discuss below.

**Figure 4:**
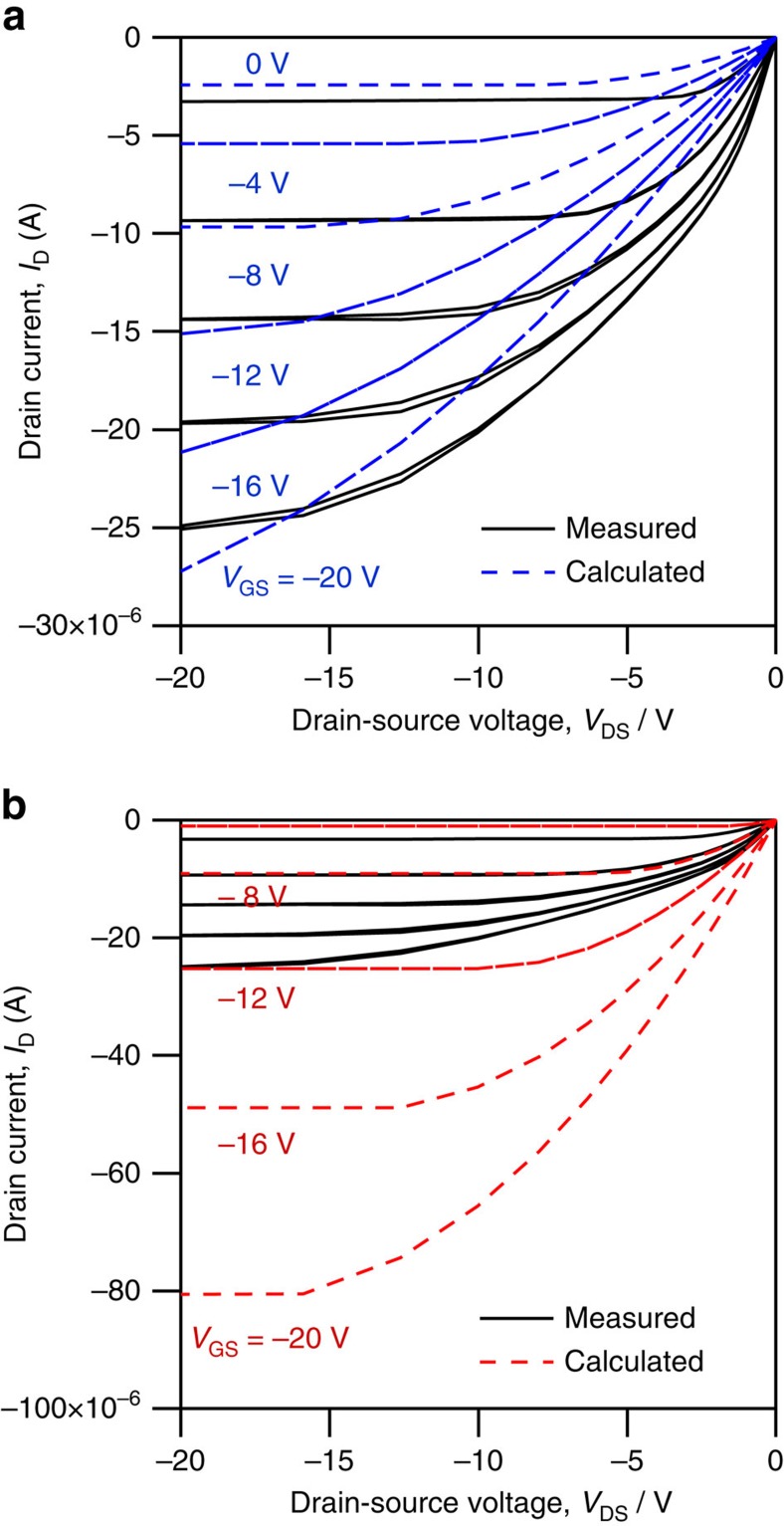
A comparison of mobility estimation results. Measured *I*_D_ versus *V*_DS_ plots compared with plots calculated from equations (1) and (2) using extracted mobility and *V*_th_ obtained from the fitting lines to the measured data in Fig. 1a for the device biased in saturation in the two distinct regions; (**a**) high gate bias and (**b**) low gate bias.

To further analyse our results in the context of the MOSFET model, we use the aggregate mobility and *V*_th_ shown in Fig. 1a for the two distinct regions to calculate the *I*_D_ versus *V*_DS_ at various *V*_GS_ by using equations (1) and (2). The calculated values for *I*_D_ (*V*_DS_, *V*_GS_) are plotted in Fig. 4a,b and reveal that the classical MOSFET relationship describing *I*_D_ (*V*_DS_) provides a poor fit when compared with the measured characteristics. The best agreement to the measured data is obtained by using the aggregate mobility (*μ*_sat_=0.9 cm^2^ V^−1^ s^−1^) and corresponding threshold voltage (*V*_th_=+8 V) extracted for high gate bias.

### Impedance analysis

**Figure 5:**
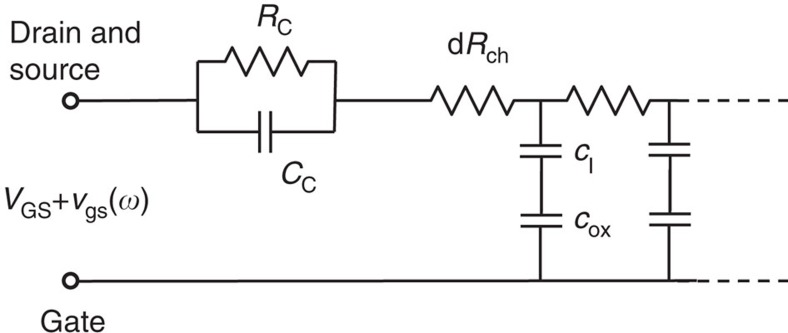
Model of the transistor impedance. Equivalent circuit used to model a.c. transistor behaviour.

We have used impedance spectroscopy to characterize and extricate the components of the transistor for the linear regime (measured at *V*_DS_=0 V) to clarify what governs device operation in the different gate bias ranges, *V*_GS_. The impedance data were analysed by using a combination of a transmission line to model the transistor channel and a parallel RC circuit to model the contacts (*R*_C_ and *C*_C_),


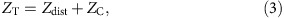



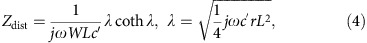



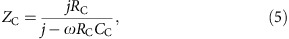


shown in Fig. 5 where *Z*_dist_ is the transistor channel impedance and *Z*_C_ is the contact impedance. The transistor channel was divided into elements of length *dx*; resistance and capacitance per area,


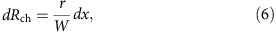



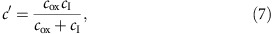


where *r* is the sheet resistance and *c*_I_ is the interfacial capacitance per area. Due to the experimental design, the contact resistance is frequency independent, whereas the distributed channel resistance is frequency dependent, and the two are easily separated. The transmission line model was shown by Hamadani *et al*.^22^ to be successful in the analysis of poly(3-hexylthiophene) transistors when *Z*_C_ is included.

**Figure 6:**
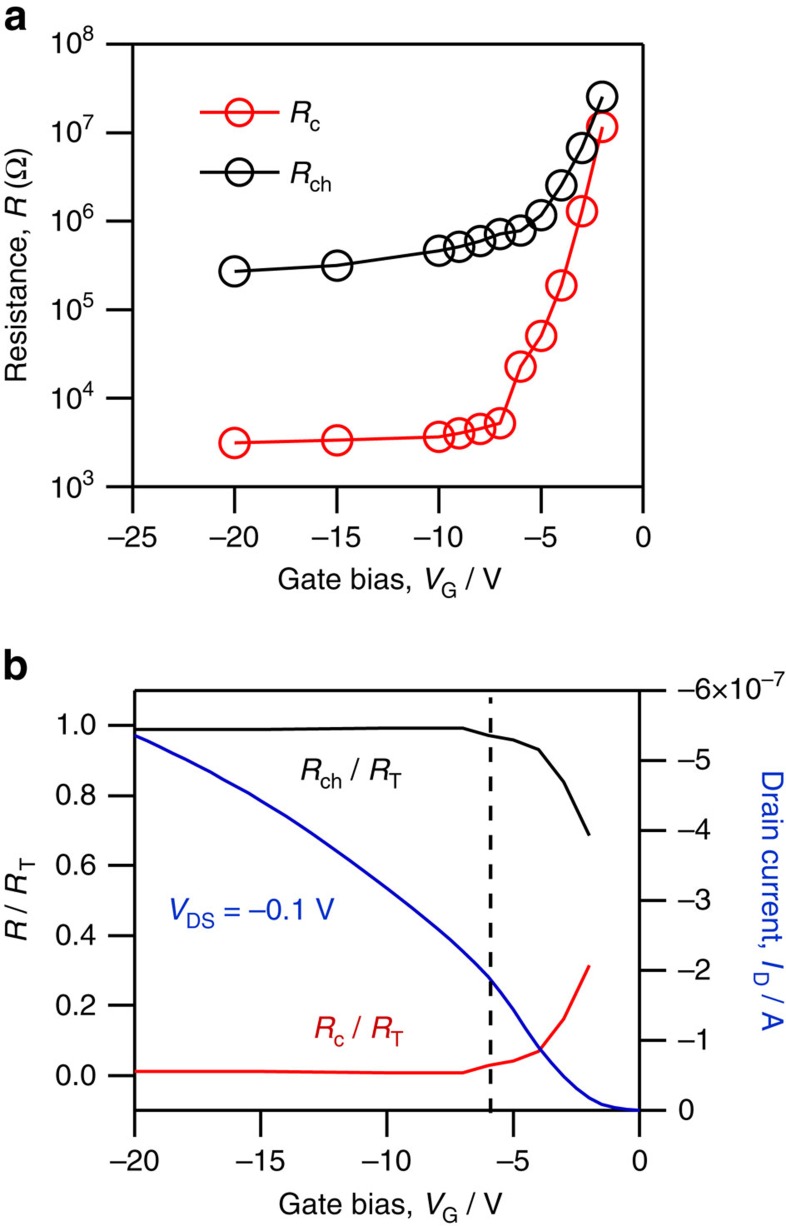
Comparison of contact and channel resistance. (**a**) *R*_C_ and *R*_ch_ values extracted from fits to impedance data using equation (3) for reverse (negative to positive *V*_GS_) sweep in the linear regime. (**b**) Plot of the I–V characteristic (blue) for *V*_DS_=−0.1 V negative to positive sweep, along with plot of *R*_C_/*R*_T_ and *R*_ch_/*R*_T_.

The channel and contact resistance were extracted for the reverse (negative to positive *V*_GS_) trace by fitting equation (3) to the impedance data and are plotted in Fig. 6a as a function of gate bias. Additional details about the impedance data modelling are included in the Supplementary Figs 3 and 4. The channel resistance varies as 1/*V*_GS_ over the entire bias range. This functional dependence is consistent with linear region MOSFET operation as given by equation (1). The contact resistance exhibits a pronounced gate bias dependence over a small bias range (0 V>*V*_GS_>−6 V), where *R*_C_ decreases exponentially by a factor of ∼5,000 as the amplitude of *V*_G_ increases. At high gate bias (−10>*V*_GS_>−20 V), the contact resistance remains at a constant low value. This functional dependence of contact resistance with gate bias is most consistent with that of a gated Schottky contact, where a relatively abrupt transition from thermionic to thermionic-field emission and finally to field emission (tunnelling) results in an exponential decrease and plateau of the contact resistance. The charge accumulation in the channel provides the necessary conditions for the tunnelling injection process and is analogous to the formation of a highly doped contact region that greatly reduces that depletion region formed at the metal–semiconductor interface and allows for efficient injection.

Figure 6b provides a graphical comparison of *I*_D_(*V*_GS_) biased in the linear regime at small *V*_DS_ (*V*_DS_=−0.1 V) to the channel and contact resistances as a fraction of total resistance (*R*_T_=*R*_ch_+*R*_C_). This comparison best illustrates the influence of *R*_C_ (*V*_GS_) on I–*V* characteristics for this device. The large change in contact resistance at low gate bias correlates with high transconductance (*g*_m_=d*I*_D_/d*V*_GS_) of the transistor *I*–*V* characteristics and with the peak in the differential mobility, Fig. 2b. At high gate bias, *R*_C_ is low (≈10^3^ Ω) relative to the channel resistance (≈10^5^ Ω) and nearly constant. In this same bias regime, we observe nearly linear behaviour of *I*_D_(*V*_GS_) and in the levelling of the differential mobility extracted from the *I*–*V* characteristics. We therefore conclude that the mobility peak at *V*_GS_≈−5 V is a result of exponentially changing contact resistance relative to the more slowly changing channel resistance.

Electrical contact between metals and organic semiconductors has long been known to have a strong influence on the operation and extrinsic performance of organic electronic devices^17^,^23^,^24^,^25^,^26^,^27^,^28^. OFET measurements of the contact resistance^26^,^28^ and local potential^14^,^29^,^30^ show that the metal–organic semiconductor interface can be a significant source of potential drop at the injection contact and can be highly influential on *I*–*V* characteristics. In particular, transistor behaviour can be significantly impacted by charge injection from the metal electrode into the OFET channel due to the large current density (10^6^ times larger than for diodes, such as light-emitting diodes and photovoltaics.) Calculations of the effects of contact resistance on OFETs, modelled by using either a Schottky barrier or low-mobility areas at the contact, show that *R*_C_ can be gate-voltage dependent, which significantly impacts the resulting current–voltage (*I*–*V*) characterization^31^.

The effect of a gated source contact on FET operation has been previously observed and/or induced in devices based on a large number of materials. For example, poly and amorphous silicon and zinc oxide FETs with Schottky contacts (source gated transistors or Schottky source barrier transistors) have been engineered to take advantage of the high transconductance and low output conductance for specific circuit applications^32^,^33^,^34^,^35^. Precisely, how the contact affects operations depends on many factors. Carbon nanotube FETs and two-dimensional layered FETs are similar to organic FETs in that conventional doping of the contact region is challenging and Schottky contacts are routinely formed to the semiconductor^36^,^37^. Injection and transport studies on the former devices^36^,^37^ report injection barriers that are typically less than 0.3 eV and a transition from thermionic to thermionic-field emission to field emission (tunnelling) with applied voltage that is less abrupt than we report here for rubrene single-crystal FETs. We expect FETs with the pronounced dependence of transconductance on bias to result from a larger injection barrier and in devices where the magnitude of the channel resistance falls within the range of the exponentially decreasing contact resistance. High-mobility organic semiconductors with contacts having large injection barriers would appear to be prone to this specific effect. Similarly, such an effect might be present but not readily observed in low-mobility organic FETs because the transition would likely occur in the subthreshold region. At a minimum, a large injection barrier is expected in devices exhibiting ambipolar operation and confirms that contacts with large injection barriers are less selective than assumed^6^,^7^.

Ambipolar behaviour is often observed in low bandgap OSCs. The poor charge selectivity of contacts that facilitate ambipolar behaviour can, under the appropriate bias stress conditions, result in electron injection and trapping. It has recently been suggested^16^ that electron trapping during bias conditions can contribute to non-ideal behaviour and give rise to over-estimation of the field effect mobility. The most likely mechanism being the current–voltage characteristics reflect non-equilibrium measurement conditions where the trapped charge is not neutralized by the injected counter charge. This permits the quasi Fermi level to move more quickly through the band gap and the current to increase more rapidly with increasing gate bias than under equilibrium conditions. Such charge trapping would only further enhance the gated contact-controlled transconductance that we report here for wide band gap organic semiconductors because of the resulting electrostatics, which inhibit compression of the depletion region at the Schottky barrier interface and efficient charge injection. The non-ideal behaviour reported for low band gap ambipolar FETs is consistent with both gated-Schottky contacts, further enhanced by electron trapping as well as measurements made under non-equilibrium conditions. A more detailed and careful study is required to disentangle such effects in these systems.

**Figure 7:**
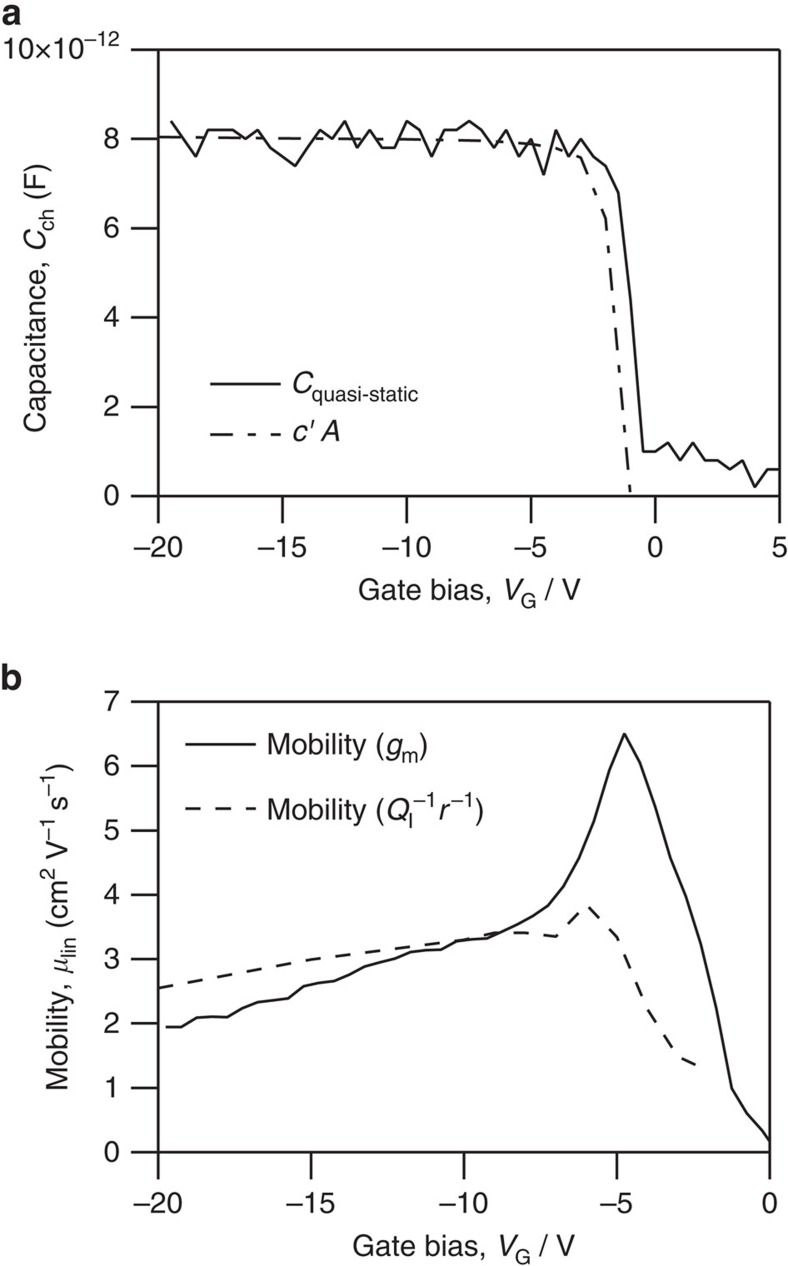
Determining the mobility. (**a**) The capacitance in the channel (*c*′*A*, where *A* is the area of the transistor). (**b**) Mobility for the reverse (negative to positive *V*_GS_) sweep: the differential mobility (solid line) calculated from the linear transistor characteristics using equation (1) and mobility (dashed line) calculated from equation (8) using the channel properties obtained from the fit to impedance data.

Although the transconductance of our transistor is dominated by the gate-activated *R*_C_ for low gate bias, the impedance measurements gave us access to the channel behaviour in this region. We can use this to calculate the true mobility of the device channel above the apparent threshold voltage of the device, which corresponds with the sharp turn-on in channel capacitance seen in Fig. 7a. Channel mobility can be calculated from the accumulated charge in the transistor channel, *Q*_I_, and the sheet resistance, *r*, in the channel by using equation (8) and the results of impedance modelling^38^. The channel mobility calculated for low gate bias from the channel *r* and *Q*_I_ plotted in Fig. 7b shows that channel mobility increases slowly over this range to a constant value and does not show the pronounced peak as in the differential mobility extracted from device *I*–*V* characteristics analysed with the MOSFET model. This impedance-based analysis of the channel mobility further supports our conclusion that the apparent high mobility is due to the effect of the gate voltage dependence of *R*_C_ on the total device transconductance and not to a variation of the transistor channel.


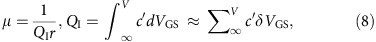


## Discussion

The importance of contacts has been widely acknowledged in organic electronic devices^17^,^19^,^21^,^39^. There exists numerous studies using gated four-terminal measurements, gated transfer length measurements and scanned Kelvin probe microscopy, all of which attempt to measure *R*_C_, correct for the reduced *V*_DS_ and *V*_GS_, and extract ‘intrinsic' channel mobility^1^,^21^,^29^. However, these approaches rely on the assumption that the measured dependence of drain current on gate voltage (transconductance) is controlled mainly by the channel properties, the channel potential as a function of position is accurately measured and the channel threshold voltage is given by the intercept of the fitting line. Furthermore, analytical expressions that model the extrinsic transistor d.c. *I*–*V* characteristics as a forward-biased diode in series with an ideal transistor do not accurately capture the physics governing device operation or the inherent two-dimensional effects of the drain and gate fields. They often yield fits to data that agree only over a limited bias range. These approaches are best suited to devices with ohmic contacts (linear *I*–*V* characteristics) that can be modelled as a resistor in series with the transistor. Efforts have been made to improve the charge injection and extraction at the metal contact to OSCs in OFETs including the use of self-assembled monolayers or contact area doping of the semiconductor^39^,^40^,^41^,^42^,^43^. When characterizing new OSCs, contact optimization is often not addressed; this can lead to over- or under-estimates of their performance potential.

It is important to note that the method detailed here for device parameterization is not entirely exempt from contact effects. Accurate parameterization near the apparent threshold voltage remains problematic because charge injection still limits channel charging and channel resistance extraction at the lowest frequency range used in these measurements (20 Hz) and results in the apparent slow increase in channel mobility as shown in Fig. 7b. At longer charging timescales and at sweep rates comparable to *I*–*V* measurements, quasi-static capacitance-voltage measurements provide yet another route to characterizing channel accumulation. Plotted in Fig. 7a is the quasi-static capacitance taken at d*V*_GS_/d*t*=0.5 V s^−1^ and showing channel accumulation at *V*_GS_≈−1 V. A small shift in channel accumulation is observed relative to the characteristics extracted by using impedance spectroscopy. Smaller voltage ramp rates are required to reveal larger shifts towards positive threshold voltage but present a significant measurement challenge. This calls into question the accuracy of other important parameters such as threshold voltage and subthreshold slope. These parameters, like mobility, are routinely used in benchmarking performance and to estimate interface trap density with the assumption that both are governed entirely by the channel interface properties.

We show here that by disentangling channel and contact impedance in working transistors, we gain a better understanding of the origin of non-ideal behaviour in the *I*_D_–*V*_GS_ characteristics of OFETs. Strongly varying contact resistance at low |*V*_GS_| results in transistor behaviour that is dominated by charge injection. This leads to an overestimation of the channel mobility by an order of magnitude when extracted using the MOSFET model and ambiguity in the transistor behaviour near the threshold. Analysis of the current–voltage data is not straightforward due to the variety of non-ideal contact and channel effects present in organic field-effect transistors. For accurate measurement of device parameters, such as mobility and subthreshold behaviour, more robust measurements and analysis must be developed along with contact engineering methods to improve charge injection at the metal–organic semiconductor interface.

## Methods

### Sample preparation

We used pre-fabricated transistor test structures consisting of a heavily doped silicon substrate (gate electrode, n-type 10^−3^ Ω cm), thermally grown silicon dioxide layer (gate dielectric, ≈57 nm) and photolithographically defined metal electrodes (source and drain contacts, 40 nm gold on 5 nm titanium). The completed transistors have channel lengths of 50–100 μm. We used a self-assembled monolayer of octadecyltrichlorosilane (OTS) to improve the semiconductor adhesion and to create a hydrophobic surface on the SiO_2_ to eliminate water^3^,^15^,^44^. This layer is assembled by immersing the prefabricated substrates overnight in 5 mmol l^−1^ OTS in hexadecane, followed by sonication for 5 min in each of the following solvents: chloroform, isopropyl alcohol and de-ionized water, and then heating the wafer to 150 °C for 10 min.

Rubrene single crystals were grown by physical vapour transport in a tube oven under argon flow and carefully laminated to the surface of the prefabricated substrates. The starting material was 99.99 % rubrene from Sigma-Aldrich and used as received. The transistors that we considered for this study were those where the rubrene crystal occupied the drain-source channel and the contacts pads only; this was done to ensure that the transistor was isolated and to remove parasitic impedance from charged rubrene outside of the transistor area, Fig. 1c.

### Electrical characterization

*I*–*V* measurements were taken with an Agilent 4155C Semiconductor Parameter Analyzer. Impedance measurements were taken using an Agilent E4980 LCR meter by applying the high potential and current terminals to the gate and low potential and current terminals to the shorted drain and source. A d.c. bias voltage (*V*_GS_) is applied to the gate along with a small a.c. signal (*V*_GS_ (*ω*)=0.025 V). At each d.c. bias point, the a.c. frequency *f*=*ω*/2*π* is swept from 20 to 2 MHz. Quasi-static capacitance was measured with a Hewlett Packard 4140B pA Meter at d*V*_GS_/d*t*=0.5 V s^−1^. *I*–*V*, impedance and quasi-static capacitance measurements were taken successively in the dark at room temperature in an N_2_ gas environment. Computer interfacing was done using Instrument Control^45^.

## Additional information

**How to cite this article:** Bittle, E. G. *et al*. Mobility overestimation due to gated contacts in organic field-effect transistors. *Nat. Commun.* 7:10908 doi: 10.1038/ncomms10908 (2016).

## Supplementary Material

Supplementary Information Supplementary Figures 1-4
