# Supplementary material for: Mobility overestimation due to gated contacts in organic field-effect transistors
Source: Nat Commun. 2016 Mar 10;7:10908. doi: 10.1038/ncomms10908 (PMC4792947; doi:10.1038/ncomms10908)
Supplement: Supplementary Information — Supplementary Figures 1-4 [file ncomms10908-s1.pdf]

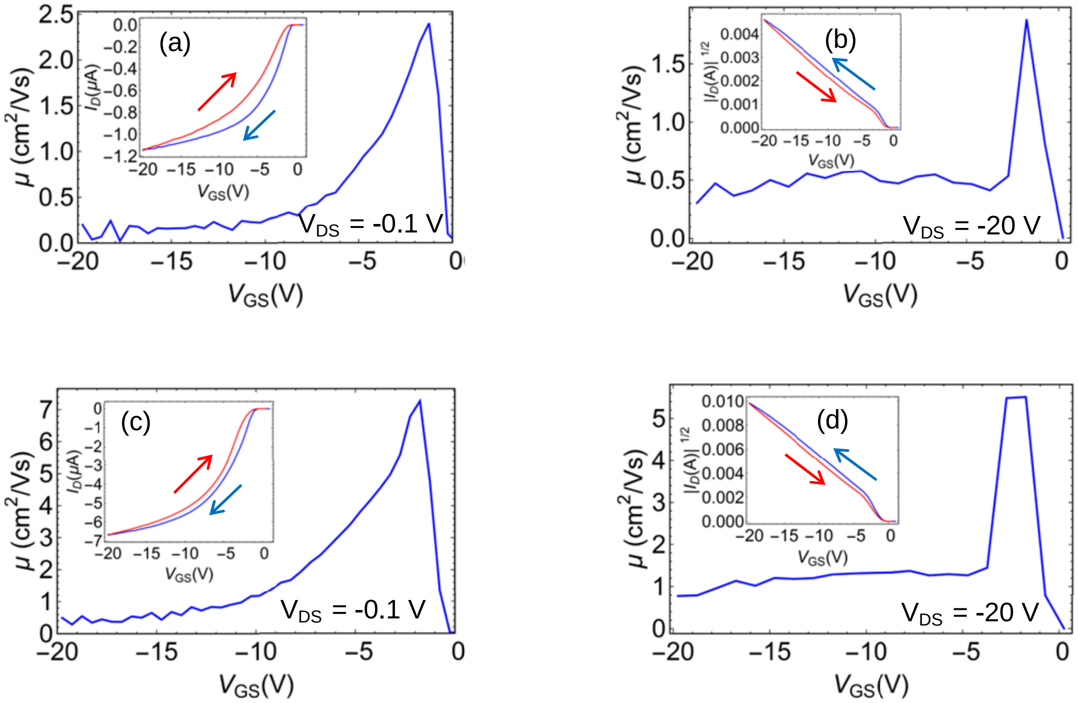

Supplementary Figure 1: **Additional transistors showing non ideal behavior** Mobility defined and extracted from Equations 1 and 2 as a function of gate voltage for linear regime (V_{DS} = -0.1 V, (a) and (c)) and saturation regime (V_{DS} = -20 V, (b) and (d)) for two rubrene transistors (a/b and c/d) with gold source and drain contacts. Insets show drain current data for each. In the linear (saturation) regime, these transistors have greater than 10× (4×) higher mobility in the low field region as compared to the average mobility between V_{GS} = -15 V and V_{GS} = -20 V.

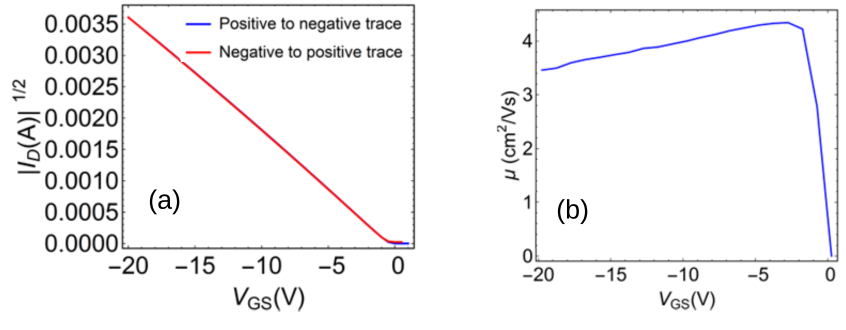

Supplementary Figure 2: **Rubrene transistor with platinum source and drain contacts showing nearly ideal transistor characteristics** Square root of drain current (a) and the mobility (b) defined and extracted from Equations 1 and 2 as a function of gate voltage for saturation regime (V_{DS} = -20 V.) Peak mobility is around 1.1× the aggregate mobility between V_{GS} = -15 V and V_{GS} = -20 V.

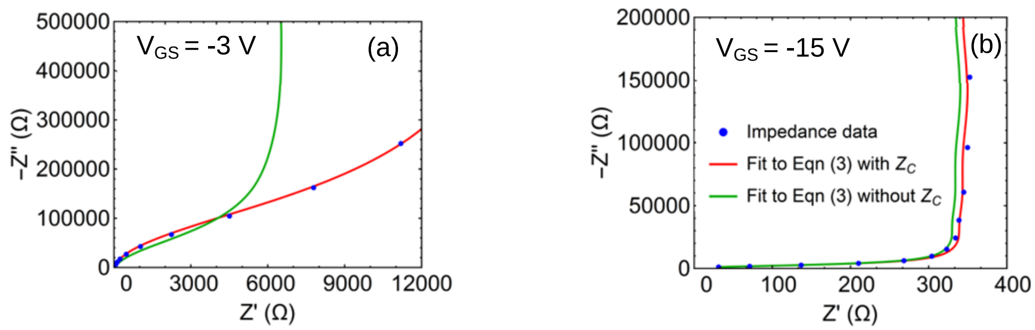

Supplementary Figure 3: **Plots of raw impedance data and fits to Equation 3** Plotted with and without contacts for 126 Hz to 2 MHz at two gate bias points, V_{GS} = -3 V (a) and V_{GS} = -15 V (b). Raw data at low gate to source voltages show that the transistor behavior is heavily influenced by the contact impedance. As the gate voltage is increased the contact impedance decreases, and the transistor behavior is then dominated by the channel.

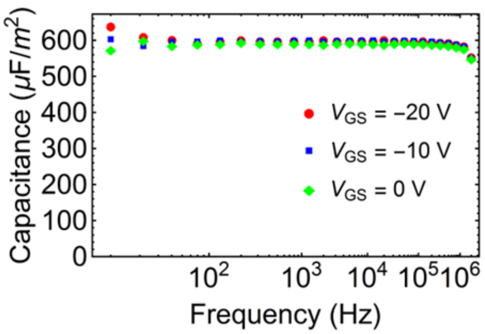

Supplementary Figure 4: **Contact pad capacitance** The large area (250×250 μm^2) contact pads add an additional capacitance to the data which is invariant with frequency and gate bias in the range measured. The capacitance of the contact pads shows a small amount of dispersion below 100 Hz and above 1 MHz. During analysis the contact pad capacitance is modelled as a capacitor in parallel with the circuit given in Equation 3.
